# Soft female leadership (SFL) framework for driving the gender equality change in engineering education: learning outcomes of leader and leadership development

**DOI:** 10.12688/openreseurope.13340.1

**Published:** 2021-06-10

**Authors:** Anastasia Zabaniotou

**Affiliations:** 1 Chemical Engineering, Aristotle University of Thessaloniki, Thessaloniki, Thessaloniki, 54124, Greece; 2 Mediterranean Engineering Schools Network (RMEI), Mediterranean Engineering Schools Network (RMEI), Ecole Centrale Marseille, France

**Keywords:** Soft Leadership; Female; Gender Equality; Engineering; Mediterranean; TARGET; Science.

## Abstract

The foundation of gender equality was built some decades ago, but higher education institutions are far from achieving it. Perhaps the leadership needs to integrate new narratives for a greater commitment especially, in engineering and new tools for the existing toolbox. This study aims to share the outcomes of soft female leadership (SFL) development for gender equality at the RMEI network, entailing the commitment of top-managers from engineering schools, creation of new leaders to lead the change at their institutions, students acting as change-agents, and an active community of practice. The SFL toolbox comprises self-awareness, humanistic care, intuition, creativity, and trust. The transformation of mindset, skillset, and culture entails using Sustainable Development Goals (SDGs), ‘Systemic View of Life’ arguments, and drawing knowledge from organizational learning frameworks, scientific phenomena, and mechanisms, such as a) The 4I-Intuiting-Interpreting-Integrating-Institutionalizin *g* organizational learning process starting from intuition to achieve an institutional change; b) The ‘Stigmergy’ scientific mechanism of self-organized collective schemes with coordinated actions and interactions, in which the action performed by an agent leaves a trace in the environment that stimulates subsequent actions; c) The ‘Spillover’ phenomenon advocating that the behavior of an agent can bring the adoption of related behaviors by other agents. RMEI gender equality plan was evaluated by the HORIZON2020 TARGET project consortium. The SFL excelled as successful in setting goals, articulating a policy that integrates systems approach frameworks, insights from science and technology, innovation, ecology, philosophy, self-awareness, ethics, and values. The Covid-19 pandemic disrupted physical meetings, but the process of change was not ceased at the network because we disrupted the disruption by boosting collaborative knowledge consolidation and dissemination processes. The SFL framework integrates context, regional, and temporal characteristics, alongside cognitive, affective, and motivational outcomes over behavioral outcomes, new mindsets beyond organizational skills, and collaborative learning over individual learning.

## Plain language summary

This paper reports a new leadership type being developed during the process of gender equality change within the homonymous Community of Practice facilitated by the Network of Mediterranean Engineering Schools (RMEI) and supported by the EU TARGET project. The type of leadership we propose is a combination of female leadership and soft competence building; that is why we call it ‘Female Soft Leadership (FSL)’. The key message is that gender equality strategies are context sensitive. We need to find for every context what problem of gender equality occurs and to make the choice of the type of leadership best fit in. This is what we did in the RMEI network that connects 28 engineering schools from 10 Mediterranean countries. Overall, leadership is a journey and not a destination and we can learn or change our way during practice.

## Introduction

Leadership in the 21 ^st^ century entails personality, behavior, and values beyond management skills, facets that are needed to face major political, economic, social, cultural, and ecological challenges ^1^. It is a collective phenomenon and social construct that shapes the way we understand organized action ^2^, which becomes more evident in the case of an unstructured group ^3^ and in navigating in disruption times, as in the on-going Covid-19 pandemic time we are experiencing globally ^4^.

Although, scientists have elaborated many theoretical concepts of leadership classifications and clarified the limits of these concepts ^5^, complex challenges of the 21 ^st^ century imply leader and leadership development approaches based on behavior-based criteria ^5^ integrating context, regional and temporal characteristics, self-awareness, and new mindsets ^6, 7^. It requires mindsets that can bring a transformation through personal and collective developmental processes ^8^. More specifically, it is acknowledged that leaders and leadership development should focus on a) cognitive, affective, and motivational besides behavioral outcomes, b) new mindsets beyond organizational skills development, c) collective over individual learning ^5, 7^.

Leaders need to adopt interdisciplinary and transdisciplinary approaches towards providing effective solutions for wicked problems of complex systems ^9^, including a clear identification of the problem, good understanding of the situation, development of a timely action plan, and good and transparent communication processes ^10^.

Soft leadership (SF) is a type of leadership that intertwines emotional intelligence with management, aiming at creating humanistic and values-based visions to build personalities transcending their known boundaries ^11^, through integrative, participative relationships, by using inspiration, collaboration, and co-creation as tools for soft skills and capacity building ^12^. We could argue that SL could be associated with female leadership ^13^.

The call for leaders to advance gender equality is an invitation to lead a wicked-systemic-social problem of which not only they must be aware, but to understand the interrelations of gender inequality with other inequalities (intersectional understanding). To be able to navigate in complexity, they must educate themselves and undergo personal developments, researching and understanding why these challenges exist and how to eliminate them by creating a culture of values and ethics ^14^.

Gender inequality is a wicked systemic problem of patriarchal societies in which women do not fully have the opportunity for personal development and decision-making that affect females lives, that is often related to stereotyped gender expectations and beliefs, and the masculine construct of leadership ^15^.

To achieve social justice, economic stability and growth, democratic governance, full participation of women in all societal processes is indispensable according to the 2030 Agenda for SDGs ^16^. Furthermore, we hypothesize that gender-balanced leadership at any level might bring more effective solutions to global challenges ^17^ while soft leadership may change the values of the anthropocentric civilization and personalities to drive the exit from the Anthropocene towards an eco-social centric development.

### Aim of the study

There is time to try to change how to make our universities more equitable, and inclusive. In achieving gender equality mainstreaming across engineering higher education, we need to rethink the type of leadership that is needed for effective outcomes. We need to add new narratives and tools to the existing toolbox and reconstruct our strategies and plans. By exploring these inquiries, we can begin to suggest where and why the journey to gender equality has stalled and how to mitigate it.

The aim of the study is to reply to the above inquiries by reporting the learning outcomes obtained from the gender equality plan (GEP) and leadership developed at the network of Mediterranean Engineering Schools (RMEI).

We report on an empirical leadership framework for gender equality that is contextual. We call it the ‘soft female leadership (SFL)’ framework that is a combination of female and soft leadership for driving gender equality at the engineering schools across Mediterranean countries. SFL is a perspective that is closely connected to a systemic view of life.

Although context-specific the SFL framework can be chosen for leading groups, institutions, organizations, etc. at any scale.

## Methods

To better understand the SFL framework and how it has impacted the learning and practice of gender equality development at RMEI and at member-institutions across the diverse Mediterranean territories, we replied to research questions. The questions we pursued in this study are:

✓ How was the SFL developed?
✓ What are the specific characteristics of the SFL?
✓ What are the impacts on learning and practicing for gender equality at individual, network, and institutional level?

### Self-positioning

***The organization: The Mediterranean Network of Engineering Schools (RMEI).*** The gender equality process we report here was undertaken by RMEI which is a network of Mediterranean Engineering Schools. It was created in June 1997 in France, and lately was affiliated to the UNITWIN/UNESCO Chair 651 on SDGs innovations (( http://www.rmei.info/index.php/fr/). 28 Engineering Schools from 10 Mediterranean countries located in Europe, Africa and Middle East are the actual active members (France, Spain, Italy, Greece, Cyprus; Maroc, Tunisia, Algeria, Egypt; Lebanon, and Palestine). Since 2018, a gender-balanced board of directors leads the network (the elect president and vice-president are women). This was the result of the SFL being followed for gender equality over the last four years, that had a very positive impact on the governance of the network.

The network’s vision is the sustainable development and a peaceful co-existence of Mediterranean people and nations in the Mediterranean basin, which is a region of high diversity. People in RMEI share a common will and commitment to the SDGs’ realization.

With the support of the EU TARGET project, RMEI is facilitating a community of practice (CoP) on gender equality (GE), inspires and supports Mediterranean engineering schools to adopt gender-sensitive activities towards a gender equality journey, especially in those Mediterranean countries that have been characterized as relatively ‘inactive’ in developing gender equality plans (GEPs). These countries are the African and Middle East Mediterranean countries where related national legislation is lacking, contrary to the European Mediterranean countries where a national GE legislation has been established due to EU efforts where they belong.

***The enabler: The TARGET project.*** The network is a partner of the four-year HORIZON 2020 TARGET project that stands for “ *Taking a Reflexive Approach to Gender Equality for Institutional Transformation*”, since May 2017. The TARGET project supports GEPs development in seven gender equality innovating institutions (GEIIs), three research performing and funding organizations and one network, the RMEI, in the Mediterranean region, by adopting a reflexive process ( https://www.gendertarget.eu/).

### The GEP of RMEI

During the last four years, RMEI has created a thematic working group, a gender equality audit (GEA), a tailored action plan (GEP), a self-assessment framework and indicators for monitoring of the GEP which underwent an evaluation in 2020 by a partner of the TARGET consortium. All interventions consisting of the coordinated activities of the RMEI GEP are the following:

- Gender equality working group (GEWG).
- Gender equality audit (GEA).
- Gender equality policy/strategy (GEP/GES).
- Gender equality policy statement (GEPS).
- Active community of practice (CoP).
- Gender equality centers/committees (GEC) at member-institutions.
- Knowledge building institutional workshops (KBIWs).
- Capacity building workshops (CBWs).
- Participatory workshops with national stakeholders (NWs).
- Michelangelo workshops (MWs) co-organized with the students of the network.
- Design of a customized self-assessment framework.
- Development of tailored monitoring and self-assessment indicators.
- Evaluation of the GEP by a dedicated partner from TARGET consortium.
- Creation of a Living Lab (LL) for knowledge consolidation, co-creation, collective reporting, collaboration, communication, and social bonds creation.
- Development of gender equality leaders.
- Development of change-agents.
- Development of the SFL framework.

### Leadership today

We can find many definitions of leadership and of the leader that are evolving over time. Leaders themselves change and therefore new approaches to leadership emerge. In past decades, leadership was the practicing of command-and-control processes. Today, leadership is driven by motivations for positive impact on people and societies. Our era is characterized by global and systemic challenges; people are called to think globally and act locally ^18^, and leaders to embrace participative, transactional, contextual, and transformational types of leadership ^19^.

Participative is the type of leadership that motivates participation by giving responsibility to members of a group/community/otherization/institution who trust the leader to take decisions ^20^.

Transactional is the type of leadership that takes clear rules and regulations, focuses on results, and rewards members practically ^20^.

Transformational leadership uses good and transparent communication, focuses on personal development, goals setting and creates a shared picture of change ^20^.

Charismatic leadership is differentiated from the transformational one because it focuses on emotional stimulation by engaging people in a shared vision. The leader is a good communicator with a positive personality. This type of leadership is more appropriate for facing complex challenges and crises ^21^.

Contextual Leadership is based on network development to bring a transformation. By using knowledge and information to guide actions, it focuses on systemic thinking and change ^22^.

Leadership for multicultural and multigeneration teams allow people to decide how to act according to their beliefs and shared values, creates leaders and change-agents ^20^, respecting the cultures existing in the CoP and creating a feeling of family and being part of the decision-making process ^23^.

### What is Soft Leadership (SL)?

Hard (HL) and soft leadership (SF) are theories and styles of leadership. HL entails strength and certainty, it adopts a ‘control and command’ approach, and it is rather effective for clearly defined simple problems ^24^. SL is a framework developed by Professor M.S. Rao in 2013 ^25, 26^ and it is appropriate for complex problems and challenges. It is based on processes of setting goals, inspiring people, encouraging teamwork, and win-win attitudes, appreciating people’s contributions in accomplishing goals and objectives. The skills that SF develops are called soft skills, entailing values and empathy under a collective mission, and shared vision ^27^. SL is differentiated from the HL since it is based on collaboration and consensus, while HL enhances competition ^27^.

**Figure 1.**
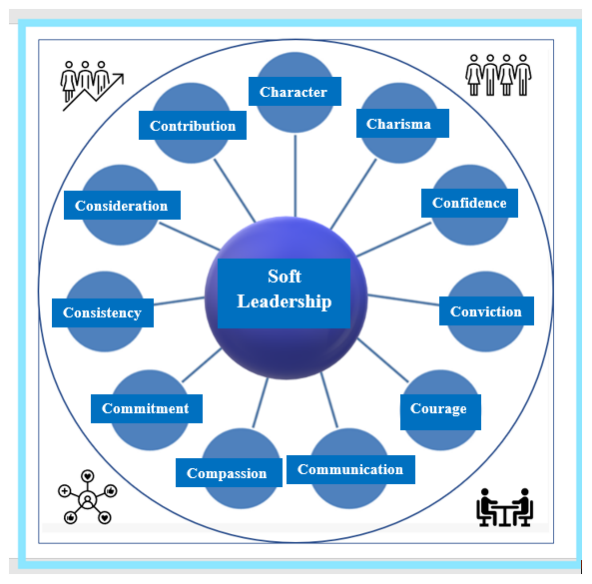
Soft leadership (SL) characteristics ^28^.

The set of characteristics of SL have been defined by the founder of the framework and include 11 characteristics (11C) such as ‘ **C**haracter, **C**harisma, **C**onscience, **C**onviction, **C**ourage, **C**ommunication, **C**ompassion, **C**ommitment, **C**onsistency, **C**onsideration, and **C**ontribution’. If six of them are met in a leadership process, then the leadership can be characterized as SL. The 11 characteristics of SL by definition ^28^ are depicted in Figure 1.

## The SFL leadership framework

### Rationale: consciously co-creating the future

We hypothesize that for a world that is changing and for designing regenerative technologies and cultures towards sustainable and equitable societies of the future, there is a need for engineering education to create new knowledge, skills, and capacities, and new leadership styles, tools, and techniques. Within this context, gender equality is a complex process.

As members of the RMEI network that regroups engineering schools and people, our main role is being educators and faculty members at engineering schools. By exercising this primary role, we do feel that engineering education plays an important role in designing technologies for sustainable societies, but we also realize that the persistent underrepresentation of women in leadership and senior positions at the engineering education systems is a challenge for realizing a sustainability-oriented education. Therefore, promoting gender equality in engineering schools and learning for sustainability that is transformative is our commitment.

Transformative learning entails inspiring and catalyzing formal and informal changes, and a shift in ways engineers think and interact with the socio-ecological systems through technological innovations. This needs engineers to be more conscious of the impacts their technological solutions have on society, to realize gender inequality in their workplaces, and take action for it. This transformative learning can come through an openness to societal needs and under new forms of knowledge and strategic networks. In networks, gender equality can be advanced through collaborative and volunteering processes that are based on willingness to change behaviors and take gender-sensitive approaches in workplace and life, contrary to the traditional rigid teacher-student flows of knowledge that govern traditional universities.

Engineering schools of the Mediterranean world are hierarchical structures led, in most cases, by male leaders with masculine perceptions on women’s position in the school, society, and family.

Therefore, for a network of engineering like the RMEI, to lead a social change that the gender equality change is, within a masculine environment, the need for a different leadership to drive that change was considered a prerequisite. A female leader to drive gender equality within the network, to facilitate a CoP across Mediterranean, and develop leaders and change-agents on gender equality was considered that fits with the RMEI’s vision and mission for sustainable development.

We also realized that not only was any female leadership needed (female leadership does not necessarily imply a different leadership), but another type of leadership with humanistic, multicultural, multigenerational, contextual, regional, and temporal characteristics. These characteristics can fit to the contextual character of engineering, to national complexities of Mediterranean countries, and to the temporal social role that engineers will play in providing technological solutions with socio-ecological benefits at local, regional, and global scales of economies.

Moreover, we realized that we must create new leaders and change agents to take the leap of gender equality in their institutions, to influence the structural institutional change in their school and workplace favoring gender-sensitive initiatives. These leaders must integrate cognitive, affective, and inspirational skills beyond organizational skills to satisfy all stakeholders.

The above reasons shaped the requirements for a different leadership development at RMEI to uptake the development of gender equality change at any level, from the individual to the working group, network, CoP, engineering schools, and engineering profession at large. All the above realizations created the foundation for our SFL framework to lead conscious individuals.

### Going beyond traditional gender lens

Our SFL framework for gender equality advancement goes beyond the traditional gender lens, which sometimes has created a fatigue in the gender equality discourse. The stand-alone argument of social justice may not seem strong enough for convincing engineers to bring the desired transformation in engineering and scientific contexts.

We based our framework on narratives and arguments that go beyond the fairness principle, for advancing the gender equality principle in engineering education, in the era of socio-ecological disruptions. We focused our arguments on the link gender equality has with sustainability and resilience to climate change hazards and disruptions, and on the synergies gender equality has with other SDGs innovations. We argued that gender equality is a critical goal for sustainable development, while its implementation can foster positive cascading effects for the achievement of all SDGs.

Our SFL framework differs from the HL framework, usually met in engineering higher education institutions that operate under hierarchies and paternalistic patterns. It differs from how information flows are distributed, how responsibilities are taken (in a volunteer base), and how willingness and commitment is endorsed.

 Our framework aims to:

- Inspire, and catalyze formal and informal changes towards gender equality, utilizing the network and external resources.
- Build knowledge and share best practices among members of the CoP.
- Facilitate multidisciplinary synergies of SDG5 with other SDGs innovations.
- Develop soft skills in facing the change on gender equality and other changes.
- Focus on the personal development of members.
- Enhance cultural change and elimination of bias.
- Emerge cognitive emotions in students and faculty members.
- Create new mindsets with emotional intelligence and critical thinking.
- Sift from reductionism to holistic perspectives.
- Sift from separation to interbeing.
- Sift from competition to co-creation.
- Shift from hierarchies to self-assembling.
- Boost responsibility and commitment.
- Facilitate intergenerational and intercultural perspectives.
- Consolidate ethics and values for a common future and peaceful co-existence in the Mediterranean region.
- Use communication across generations.
- Use communication across different cultural and national contexts.
- Develop change-agents for gender equality.
- Develop leaders.

### Multilevel of gender equality learning

The framework aims for individual (personal) and collective capacity building for gender equality. Knowledge building, skills development, and attitude changing can improve the individual's capacity to proceed with a gender equality change. Personal development refers to the psychological processes that occur in the individual through learning and acting that can influence changes at the collective level via interactions the individual has with other members of the collective entity (working group, network, CoP etc.) ^29^, and through the ‘Spillover’ phenomenon. The ‘Spillover’ phenomenon was first explored in chemistry ^30^. But it can be observed in human groups behavior where one individual behavior can cause the adoption of related behaviors by others ^31^.

**Figure 2.**
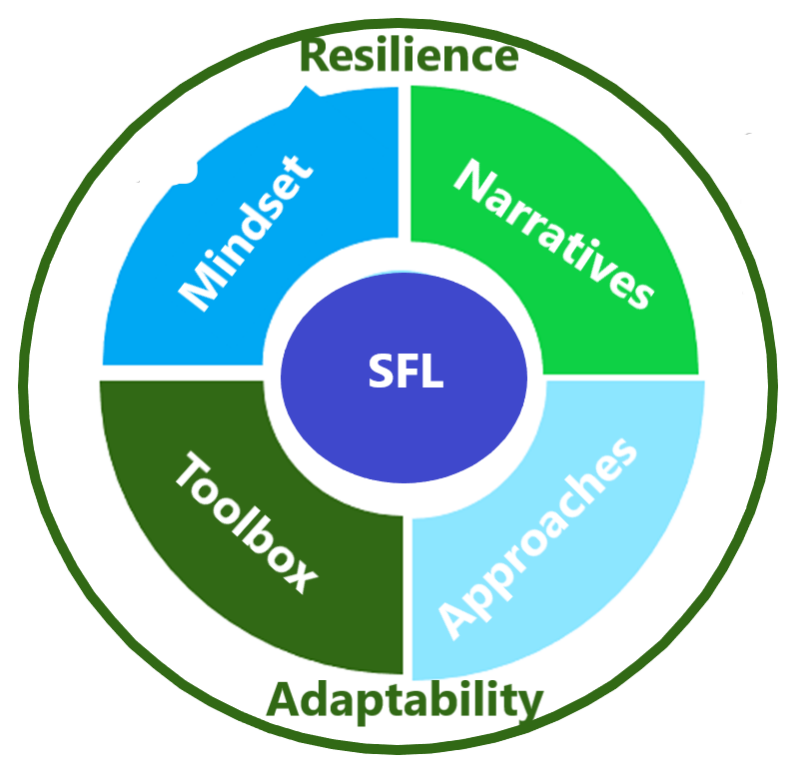
SFL framework’s new deliveries.

In the following paragraphs we present the SFL framework characteristics. Figure 2 depicts the SFL new deliveries that include narratives, approaches, toolbox, and mindset, and considers adaptability and resilience in the process.

***Step 1: SFL new narratives***

 We assumed the need to change the narratives around gender equality in engineering education settings and went beyond a charity cause, since we observed that the charity cause cannot be relegated to the realm of social justice within technocratic settings ^32^. Therefore, we brought to the discourse new arguments and discovered new narratives that can shed light to the multifaceted gender equality principle, while we explored other promising frameworks for transforming higher engineering education towards this objective.

Since we recognize that gender equality is a wicked problem, inherent in any socio-economic system, as well as in higher education systems, we integrated arguments based on natural and social capitals, ethics, and values, to serve as agencies for planning and gender equality mainstreaming, advocating that these integrations can accelerate fundamental changes towards a sustainable, equitable and resilient life.

The key message is that our framework is based on new narratives that can help overcome social, contextual, and territorial assumptions and difficult settings, such as the engineering ones. Our narratives can foster systemic gender equality and holistic approaches for a whole-system transformation.

***Looking beyond traditional gender lens.*** Gender lens approaches have expanded in recent years regarding the implementation of gender equality in universities/organizations. The aligning of the traditional gender lens with the SDGs gains a broader perspective. This alignment matters because it facilitates the practice of gender mainstreaming in engineering settings. Gender mainstreaming matters because it acknowledges that all SDGs (from climate change to food security, resources, energy) impact different genders differently.

Going beyond gender lens and fairness argument, we advocate that since gender equality is a challenge for many sectors, (from education and research, to production, economy, etc.), it has implications with climate change, health, security of food, water, waste, energy, biodiversity, and technological innovations. We argue that the transition towards socio-ecological resilience should be aligned with the fundamental aspect of gender equality and that by increasing gender equality in all sectors a positive impact on productivity, innovation, sustainability, resilience, and democracy can be brought.

Differentiated vulnerabilities and gender inequalities can impede the effectiveness and sustainability of climate change responses which are essential for tackling global challenges.

The key message here is that women equal participation in all sectors should be a contextual policy and a leverage for sustainability, resilience, and democracy.

***Gaining a contextual perspective.*** Engineers can play an important role in most of the SDGs’ implementation, mainly by designing cost-efficient technological projects to preserve natural resources and the environment. In the 21 ^st^ century, engineering is defined as the ‘social practice of conceiving, designing, implementing, producing, and sustaining complex eco-socio-technological products, processes, or systems’ ^33^. We believe that technology must serve the needs of humanity, in terms of providing sustainable resources for the present and future generations ^34, 35^.

A good leader in engineering settings and sensitive to gender equality principle can find synergies of SDGs technological and engineering innovations with gender equality and identify R&I activities to connect with gender vulnerability agency (to connect gender equality with clean water and sanitation systems (SDG6), energy systems (SDG7), infrastructures (SDG9), cities (SDG11), production and industrialization (SDG 9, 12), and in climate action (SDG13)).

The message here is that, when augmenting gender equality in engineering, it is basically arguing for sustainability and sustainable development implementation, because SDG5 should be horizontally integrated into all SDGs. Any top manager of engineering context and setting, committed to sustainable development and having understood the interconnectivity of all SDGs, cannot deny the commitment to gender equality that is crucial in accelerating sustainable development.

***Gaining a complex systems perspective.*** SDGs implementation requires solutions that need critical thinking, creativity, and knowledge-transfer. It requires to take a complex system approach that integrates social sciences, engineering, and humanities ^36^.

Systems thinking is a useful tool in addressing complex situations. It is problem-solving framework that explores inter-relationships of the system, perspectives of the situation, and defines critical boundaries ^37^, while it enables a more contextual perspective by shedding light on the role of social, economic, and historical conditions ^38^.

Engineering institutions should recognize that gender equality requires changing the entire system, because gender equality is nested within the system, and they need to adopt a whole-system approach from leadership to capacity development, accounting, and assessment of the institution to creating a new wisdom ^39^.

The message here is that an appropriate leadership for gender equality must take a systemic and contextual analysis of the problem, reflexive, and circular approaches, to go beyond reductionism that claims individuality in society without interconnections ^40^.

***Reminding the social role of universities.*** The sustainability-oriented education and transformation at the higher educational level calls for equality and diversity ^41^. In the 21 ^st^ century, universities are the key drivers of education for sustainability (EfS). They have the responsibility to integrate gender equality into teaching, research practices, and to bring a shift in ways of thinking. Engineering schools have the responsibility, besides developing appropriate competencies in designing technological products, processes, or systems, to raise awareness for sustainability and inclusivity ^42, 43^, and developing leadership capability ^44^.

The message here is that when augmenting for gender equality in engineering settings, it is basically augmenting that both men and women engineers, managers and scientists should make substantial contributions to the sustainable development, work equally on sustainable solutions that benefit entire societies. Gender equality can boost an interconnected and knowledge-based world economy that can distribute equal opportunities for men and women.

***Step 2: SFL adopted approaches***

***The STIGMERGY mechanism: self-assembling systems.*** The science based ‘Stigmergy’ mechanism can be useful in addressing complex problems by self-organized collective schemes, with coordinated actions and interactions of individuals, and feedback ^45^. It is a biological term documented by Pierre-Paul Grassé (1959) describing the mechanism of termites to coordinate actions without having a management structure ^46^. This mechanism as a phenomenon can be applied to human environments, both natural and engineered, and it is especially referring to CoP and networks’ coordinative contributions ^46^. In human systems, it means that individuals actions leave traces in the environment that affect the behavior of other individuals in the network ^45^, and changes that occur in one part can influence changes in another part of a system ^47^.

The message here is that in a CoP or a network that are self-organized collective schemes, with coordinated actions and interactions of individuals and feedbacks, gender equality change occurs when one member actions affect subsequent behavior of other members, creating the so-called change-agents.

***SPILLOVER phenomenon: participatory systems.*** As scientists in chemical engineering, we are aware of the ‘Spillover’ phenomenon that defines effects of an activity spread further than was originally intended ^48^. We assumed that for a gender equality perspective in engineering education, spillover could happen as an interesting phenomenon to shed new light on how the process of a change occurs, and how an individual can become a change-agent ^49^.

Gender roles are learned through social processes and have a strong impact on the gender progress in Universities. The spillover behavioral phenomenon applying on a system composed by many entities like the RMEI network (that is composed by different engineering schools), can help in developing GEPs, based upon a common willingness to share, and extend experiences beyond the context of our initial community in which we work. This can result in the following: one well-performing school (member of RMEI) in one country can inspire another school to do the same in another country, regarding the development of a GEP, and to adopt it accordingly to the specificity of national context. Therefore we hypothesized that, by sharing common values of sustainable development and equality in a clear, open, and collaborative way to the members of the network (professors and students), we could catalyze gender equality practices in all engineering institutions of the Mediterranean CoP, based on the “behavioral spillover” which is the notion that “one behavior triggers adoption of other behaviors”.

The message here is that evaporation of some biases can be catalyzed through trustful interrelations, interactions, and interchanges among members of a CoP and due to the behavioral spillover phenomenon, which takes place where the adoption of one behavior causes the adoption of additional related behaviors by others.

***The 4I organizational approach (Intuiting, Interpreting, Integrating, Institutionalizing).*** The ‘4I’ approach of organizational learning describes the processes that are involved in creating, retaining, and transferring knowledge within an organization/institution, that can be applied at many scales. According to the 4I approach, the process of learning is taking the steps of Intuiting, Interpreting, Integrating, and Institutionalizing. Interpreting is a more conscious process than intuiting, involving conversation and dialogue that leads to mental shift among individuals, while integrating involves taking coordinated actions and focusing on collective action. Finally, institutionalization occurs when new ideas and actions become embedded into rules, procedures, and infrastructures ^50^.

The message here is that in conceptualizing the gender quality change at the RMEI network and member-institutions, we took the steps of:

• Intuiting (at faculty staff and students’ level).
• Interpreting (bridging faculty staff and students with the gender equality working group).
• Integrating (shared understanding among members).
• Institutionalizing (at the network and member-institutions level).

***Step 3: The SFL new toolbox***

Once we positioned SFL as a driver of gender equality change, we provided the tools that enable the change to thrive.

***A clear vision for a desirable future based on common values.*** Willing to bring about gender equality change in the network we created a clear vision of the type of change we wanted. To change a culture of leadership we were based on mutual respect and trust, empowering others to become ‘change-agents’ at their institutions, as well at their life.

The SFL leader must compel a strong set of common values among members and create relations that are based on trust. The adoption of a GEP statement was the result of members’ commitment to common values. GE is an ethical concept, with values and beliefs underpinning it.

The new toolbox offers the discovering of own values in the domain of sustainable development shared, with other individuals and the collective.

***Interdisciplinary and transdisciplinary approaches for systemic solutions.*** For engineers to solve complex socio-ecological problems, there is a need for interdisciplinary approaches beyond technological knowledge and simulation models. Sensibility to interdisciplinarity, transdisciplinarity, and complexity matters for a global transformation process ^51– 55^.

In a disciplinary approach to a problem, each scientist uses processes to solve the problem through a developed methodology. For example, science and engineering contributes technical and scientific innovations, while social sciences contribute language and ethics ^52, 53^. In an interdisciplinarity approach a connection of both fields of knowledge is occurring ^7^.

SFL tool is encouraging to work closely with experts from social science, humanities, art, and other stakeholders, to overcome the barriers of gender equality in engineering education.

***Creativity in solving complex problems.*** Problem-solving learning is particularly reflected in the process of engineering education. Solving a problem is a complex cognitive process requiring mental effort, critical thinking, and creativity ^55^. The realization of problem-oriented learning requires to develop skills of critical thinking, skills in argumentation ^56^, while various contextual factors impact the creative process of informal social groups ^57^.

The SFL toolbox creates capacity and openness to support creative ideas in the process of innovation or change. In an ever-changing world to meet the needs of societal demands, creative leadership and critical thinking are required, which SFL offers.

***The Pythagorean perspective: art-based learning.*** Literature, music, mathematics, and art are constituents of the Pythagorean perspective ^58^. Learning involves enriching the ‘Self’, broadening visions of an individual ^59^.

Art-based learning can be used in gender equality CoP-based projects to connect to social inquiry and foster a dialogue ^60– 62^, developing connections between different ways of knowing and interdisciplinarity ^52^, allowing different opinions to be expressed with a nonviolent communication ^62^.

The art-based methodology is what RMEI leaders use during the Michelangelo Workshop, annually co-organized with the students of the network (GAMe sub-network), for gender equality learning.

***The Socratic “know yourself”: developing character and consciousness.*** First, Socrates, the Greek Philosopher, with his famous ‘Know Thyself’, has advocated working on yourself to obtain consciousness ^63^. Similarly, in China, *Confucius* approached learning and education by emphasizing character cultivation of individuals ^60^.

The concept of consciousness appears in the field of psychology and it is defined as self-awareness, awakening state, and knowledge, according to Velmans (2009), entailing a system of beliefs. It is the state of an individual being aware of something, and it is related to individuals' tendency to engage in social change activities ^64^. It entails:

• Emotional dimensions (beliefs and values).
• Perceptions (attitudes).
• Cognitive factors (information and knowledge).
• Action taking (environmental behavior)

Consciousness can be referred to as the experience of the 'Self' ^65^ while understanding how human consciousness is functioning can deliver societies with significant opportunities ^53^. Workshops can help consciousness enlarging and understanding of gender bias and biased conceptions ^66^.

The SFL toolbox is used within the RMEI CoP in helping professors and students understand the relationships between their actions and the impact they have on society and others. For this reason, awareness workshops and symposiums were organized by RMEI leaders.

***Einstein’s quote ‘the intuitive mind is a sacred gift: emerging intuition.*** Albert Einstein has had often quoted intuition with ‘the intuitive mind is a sacred gift’ *.* Some other researchers have been debating the value of intuition in decision-making for years ^67^, while cognitive scientists accept that intuition can lead to effective decision-making, as Gerd Gigerenzer from the Max Planck Institute for Human Development in Berlin, argues in his book entitled ‘Intuition is the Intelligence of the unconscious’ ^68^. Capra (1989), in his book entitled ’Uncommon Wisdom’, argues that humans dispose an extraordinary reservoir of power, love, and wisdom within ^69^, that needs to be activated by practices ^66^.

Intuition can be a helpful tool. This is a SFL tool used within the gender equality working group (GEWG) in shaping the GEP for RMEI.

***Step 4: SFL new learning processes***

***Introducing new forms of learning: living lab.*** For a gender equality change new forms of learning (like the living lab) can benefit from the co-sharing, collaborating conditions of a network by engaging stakeholders actively ^70, 71^. Living lab for gender equality is a transdisciplinary form serving as the vehicle and offering space for systemic change activities ^72^.

A living lab has been organized in RMEI to be the vehicle for gender equality systemic change, offering space for collaboration and innovations. The living lab was very effective for the RMEI members during the disruption that Covid-19 pandemic has brought with closed borders and online communication, because it offered the virtual space for interacting and collaborative papers writing (14 collaborative papers collaboratively submitted to peer review journals) for the consolidation of knowledge in gender equality.

***Introducing transformation.*** For a gradual transformation of engineering education institutions, fostering awareness of the ‘systemic view of life’ can enhance individual change ^7^, making a shift from bias perceptions into gender equality in co-existence ^4^, adopting finally a ‘whole-institution approach’.

We believe that transformative learning deserves a role in engineers’ education to go together with technological achievements, inventions, and innovations.

***Step 5: SFL new mindset***

***Affording the heterodox views, respect democratic freedom, reaching a consensus.*** The concept of gender equality is a cross-cutting issue of social and cultural constructions on women and men’s social relationships ^73^. Various cultural representations have a strong influence on gender equality beliefs and perceptions ^74^, acquired through social processes ^75^. Cultural change in a geographical context is seen as a change in shared values ^76^.

In developing a gender equality policy (GEP) at RMEI, we had to respect the high diversity of national contexts, without demonizing heterodox conceptions and beliefs. To transcend the complexity of national contexts, we used the arguments of ethical commitment to SDGs, common values, trustful relationships, and effective communication.

Our SFL framework is respecting national-context complexities existing in Mediterranean countries, depending on the level of gender equality advancement in each country, knowing that this differentiation can also create differentiation in perceptions of individuals on gender equality processes and level of interventions for RMEI. We overpassed these difficulties by respecting the cultural differences, heterodox views and disagreements among members, trying to always reach a consensus.

***Avoiding judgmental comparisons.*** Gender equality change asks for voluntary actions. People who are participating in CoP are volunteers; they offer time and energy for the CoP that is beyond their professional workload and obligations at their institution.

Among members of the RMEI, gender equality policies and institutional ambitions are more advanced in some institutions, mainly from European countries where European and national legislation is in place. More ambitious policies and new gender equality options are needed for the less-advanced ones.

 SFL calls for co-creation on a volunteer basis and for exchanging knowledge and best practices, while avoids creating a competition among the low-advanced individuals and institutions with those that are high-advanced in gender equality practices. It empowers by inspiring members to enhance their commitment to SDGs, encouraging the use of gender equality strategies in a way that does require a deeper theoretical understanding of the change theory, but a practitioner character that fits to engineering settings, and making optimum use of network’s capacity.

***Need for responsibility and commitment.*** A leader for gender equality is someone who can inspire through the formation of positive relationships and empowering others to lead ^13^, inspiring initiatives to strengthen consciousness, responsibility, and commitment that then can give birth to new gender-sensitive initiatives. Responsibility assumed by the members of the network can provide a commitment and a sense of the mission ^77^. Trust must be sustained with evidence-based interventions and transparent communication. 

SFL inspires humanistic approaches, improves individuals’ responsibility, creates trustful relations, stimulates emotions.

***Credibility in communication with intergenerational, intercultural, international groups.*** For leading multiple generations from diverse cultural backgrounds (faculty members and students from 10 different Mediterranean countries), we took into consideration the different strengths and weaknesses of each sub-group, interchanging with them with respect, challenging them to contribute to the CoP by finding ways to involve them in initiatives and processes, in a win-win basis.

The RMEI CoP is a multicultural and multigeneration group; members choose their actions according to their beliefs and experiences.

***Consolidating knowledge and dissemination of results.*** It is important to consolidate knowledge on gender equality and disseminate the results. During the Covid-19 lockdowns, we achieved knowledge building and consolidation by writing collective publications on:

- Statistical analysis of gender segregated data from various member-institutions.
- Questionnaire building and analysis of responses on perceptions of gender equality in Mediterranean Engineering Schools and society, and on other domains of engineering innovations and practices.
- Analysis of responses on Covid-19 remote work impact on Mediterranean female researchers.
- Resilience to climate change hazards and implication of gender differentiated vulnerabilities.
- Self-assessment frameworks and monitoring indicators development.
- Lessons learned.

A SFL leader is a person capable of finding ways to keep the community active. In RMEI, we provide capacity, experience, guidance, and creative ideas for knowledge consolidation by writing collaborative publications for peer review international journals and for conferences, to collaboratively disseminate results on gender equality.

***Step 6. Resilience and adaptability-disrupt the Covid-19 disruption.***

***Lockdown and online remote work.*** The face-to-face aspect of CoP and university life was disrupted during the Covid-19 lockdowns. Meetings, conversations are ceased. It is showing that the socio-economic implications of Covid-19 are impacting women disproportionately ^78^ because the increased childcare during schools closing is having impacts on the distribution of home activities and childcare ^79^. Addressing the question of how the current Covid-19 pandemic reveals existing gender injustices, we propose new caring social norms to universities ^80^.

***Digital stage.*** In the Covid-19 disruptive age, physical services are being replaced by online ones, reducing the interventions and activities of our CoP ^81^. However, the current transition to the digital stage provides an opportunity for:

- Redesigning learning processes on gender equality.
- Aligning technology and gender equality skills.
- Consolidating knowledge.
- Building human-centric leadership culture.

RMEI adapted to the Covid-19 disruption by keeping the CoP active via introducing new ideas of activities for knowledge consolidation, inspiring, and supporting collaborative publications on gender equality issues. In addition, collaborative surveys with dedicated questionnaires on how the Covid-19 remote work has impacted Mediterranean female academic developed and distributed to Engineering Institutions faculty members and young research, PhD students etc. while the outcomes the foundation of publications sent to international journals and other Universities networks. This is an on-going process. The above increased the adaptability and resilience of our GEP and CoP.

To increase resilience and adaptability and strengthen the feeling of belonging to a CoP that can be weaken especially for those who joined the CoP recently or due to the lack of face-face interactions during Covi-19 pandemic, we countered that by keeping an active online communication and organizing capacity building workshops online.

## Discussion

The SFL and leaders’ development framework for gender equality advancement in engineering, that we developed at RMEI, is grounded on empirical evidence. It derives as an outcome from an engineering context across Mediterranean countries.

We countered the lack of knowledge on change theories, by using our intuition, creativity, interdisciplinarity approaches, and our embedded experience from scientific and academic work, engineering problem-solving methodologies and R&I approaches. All these complemented with tailored and customized activities, under a reflexive prism that the TARGET project taught us.

The new toolbox and mindset enabled by the TARGET project, during the years 2017–2021. The RMEI GEP has been evaluated by the consortium of the TARGET project. SFL excelled in the evaluation as ‘very successful’ in setting goals, articulating a GEP, and facilitating a vivid CoP, inspiring institutional changes, and endorsing the commitment of top managers of member-institutions.

SFL constructs new narratives for a gender equality dialogue in engineering context, that is based on SDGs commitment and systems frameworks, and draws insights from science, technology, innovation, ecology, philosophy, ethics, and values.

The SFL model integrates multidimensional, context, regional and temporal characteristics to create cognitive, affective, and motivational over behavioral outcomes, new mindset beyond organizational skills, and collaborative learning over individual learning.

SFL is an innovative framework for leader development because it goes beyond the traditional gender lens which sometimes create a fatigue in the engineering education discourse, not a strong enough argument to stand alone for the engineering and STEM specific context.

SFL narratives link gender equality to sustainability, show synergetic effects with all SDGs innovations, gender equality as the critical goal for sustainable development and it demonstrates how its implementation has positive cascading effects to all sectors.

SFL combines female soft leadership characteristics; it is very appropriate to advance gender equality in engineering and STEM contexts and settings. Female leadership alone does not guarantee that it is not a hard leadership (HL), while the combination of female and soft leadership (SFL) might be the best for advancing gender equality at all contexts and levels.

**Figure 3.**
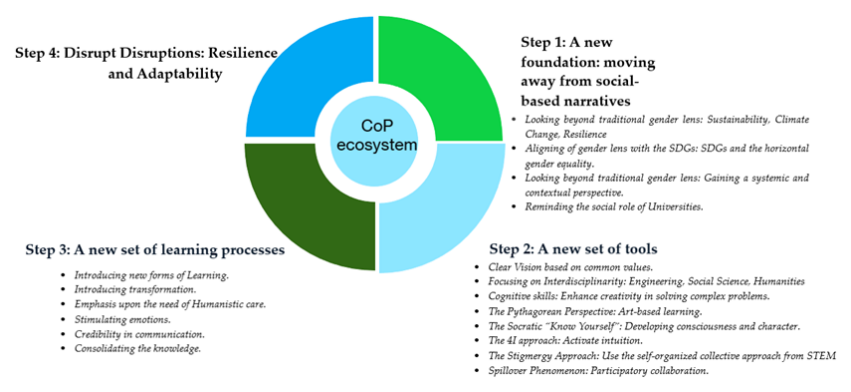
SFL and leaders development steps.

Figure 3 shows the steps of SFL development with the CoP of RMEI.

**Figure 4.**
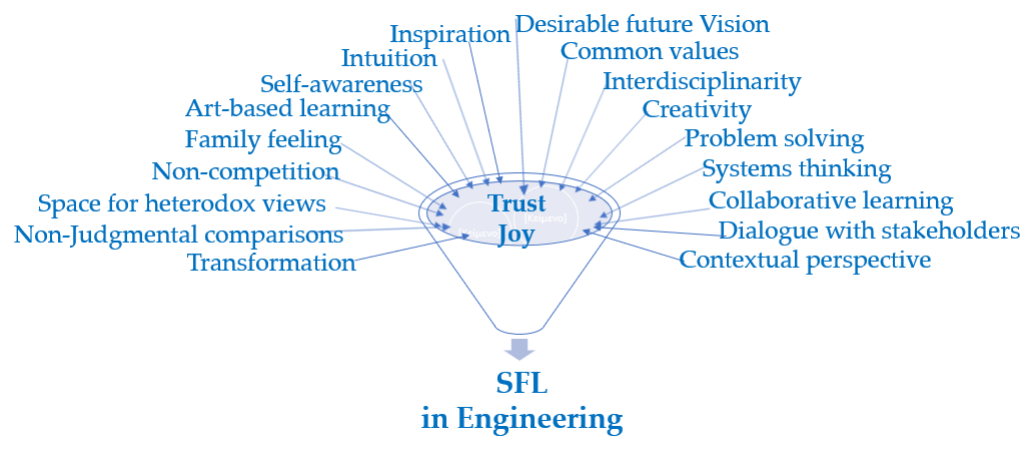
SFL characteristics for the engineering education context.

The characteristics of SFL for gender equality advancement in engineering context are depicted in Figure 4.

The key point for the transformation, that SFL opts for, is the understanding of our powerful interconnectedness and interdependence as a fundamental property of an expanding transforming and evolving universe. Once people have understood the above, they become conscious of their own responsibility to co-design and co-create a healthier and more equitable future. This awareness of our co-creative agency is based on the shift of how we see things, from the mechanist and reductionist perspective of an objective observer to the holistic and ‘systemic view of life’ of a subjective participant. This is a shift in narrative from the cultural dominance of separation to interbeing, equality at all levels and co-existence with peace ^82^.

## Conclusions

There are multiple types of leadership. In this paper, the soft female leadership (SFL) framework for gender equality change in engineering context is reported.

SFL is a leadership framework combining female leadership and soft skills, focusing on the ‘Systemic View of Life’ concept.

SFL is a leadership for the 21 ^st^ century challenges that implies multidimensional, context, regional and temporal characteristics, provides cognitive, affective, self-awareness, and inspirational tools for the creation of new mindsets beyond organizational skills, and collective learning over individual learning.

SFL framework for gender equality advancement goes beyond the traditional gender lens which as standalone can create a fatigue in the education for the engineering and STEM contexts transformation. Awareness of the systemic view of life and emotional intelligence along with humanistic values are included in SFL as agencies for gender equality actions (theory and praxis).

SFL is a combination of female and soft framework that reflects multiple dimensions for individual and collective change; it was used for building GEPs at the RMEI network and members.

Female leaders taking a soft leadership approach may be the best for advancing gender equality at an engineering context.

The outcomes of this study and the SFL model for leaders’ development to drive gender equality within masculine-dominated engineering institutions and complex national contexts, may inspire other institutions and sectors to adopt it.

The key message is that gender equality strategies are context-sensitive and thus there is a need to discern what problem of gender equality occurs in every setting, and to make the choice of leadership best fit in.

## Data availability

The author confirms that the data supporting the findings of this study are available within the article.

## Ethic statement

The author confirms that no additional ethical approval was required for this study. TARGET formulated ethical guidelines in the course of the application and in the form of deliverables were approved by the Ethical Committee and the project was not required to undergo a full ethical review according to EC regulations. The paper reflects the authors' own research and analysis in a truthful and complete manner.
