# Supplementary material for: Learning outcomes of leader and leadership development: Method of Soft and Female leadership (SFL) empirical framework for driving the gender equality change in Mediterranean Engineering Education
Source: Open Res Eur. 2021 Oct 13;1:63. Originally published 2021 Jun 10. [Version 2] doi: 10.12688/openreseurope.13340.2 (PMC10445827; doi:10.12688/openreseurope.13340.2)
Supplement: Peer review history

# Peer review: Reviewer response for version 2

**DOI:** 10.21956/openreseurope.15212.r27799

**Published:** 2021-10-21

**Authors:** Elizabeth Pollitzer

**Affiliations:** 1 Portia gGmbH, London, UK

I thank the author for responding to the earlier comments. The background, purpose and content of the paper are now more clearly connected to the work done during and the experience of the TARGET project. In this case, I support the indexing of this report. The realisation that came to me on reading the revised version is that I am still not fully comfortable with the term "female leadership" but understand that this comes from the TARGET project so it is appropriate for this paper. However, I would recommend that the author considers the term "inclusive" when disseminating this work in the future. Firstly, because many (men *and* women) science and technology leaders today are struggling with what inclusivity in organisations and organisational excellence mean and the proposed framework helps provide answers to that. Also, from the perspective of GEPs implementation, the term "inclusive leadership" may help produce more positive response to GEPs as tools for change  in engineering institutions than "female leadership".

Reviewer Expertise:

gender equality and gender dimension is science research

I confirm that I have read this submission and believe that I have an appropriate level of expertise to confirm that it is of an acceptable scientific standard.

# Peer review: Reviewer response for version 2

**DOI:** 10.21956/openreseurope.15212.r27800

**Published:** 2021-10-20

**Authors:** Jerneja Penca

**Affiliations:** 1 Euro-Mediterranean University, Piran, Slovenia

The author has made considerable effort in addressing the comments by the reviewers, including mine. The "method" article seems to be a more appropriate type for this manuscript.

 I am still struggling with a key aspect - understanding the value added of the present piece. I understand the author wants to focus on presenting the approach of their group to gender equality, but we are not given an explanation as to a) why this needed to be developed and b) what is element that differentiates the suggested model from the approaches that have been discussed in the past. The article mentions that engineering education in various places has been examined but we get very little information on that: what are the obstacles, circumstances to which women are introduced? A value-added of the transnational network is precisely the access to multiple national practices. Unfortunately, these are hardly presented, observations/conclusions are hardly shared. Instead, the article focusses on re-introducing some approaches (through the established jargon), assembling them in a new way and introducing a new "model". More bluntly, what is the advantage of the new model (and the value-added of the piece) over stating that soft, locally specific leadership is important?

 I trust the consortium has gathered a number of interesting experiences, narratives, stories that would provide necessary layers to the gender debate and make it a lot more nuanced. My suggestion would be to try to make them explicit & spell out the real life circumstances in order to underpin the debate and have us understand why is the new "SFL" needed at all. We are lacking justification to the proposed model - it could be based on lit review or the experience encountered in the network. Even if the article is about introducing a new methodology, proposing a new methodology needs to be based on justification of why it is needed or an awareness of the practices out there.

 The writing style is still not conducive to having a reader read a narrative. One sentence paragraphs create a very fragmented narrative.

 Also the abstract is still difficult to read. I suggest to considerably improve its readability. A lot of methods are introduced, but it is still not clear which ones are new (developed by the new method), and which ones not. Also the acronym RMEI is not defined. There are various instances of very unclear language, e.g. "the process was not ceased ... because we disrupted the disruption by boosting ..."

 My key suggestion for improving the piece would be to embed the article either in the existing literature, or (perhaps preferably, given the ambition of the consortium), the knowledge and experience about leadership that exists within the experience consortium and was probably highlighted in the course of the project.

Reviewer Expertise:

NA

I confirm that I have read this submission and believe that I have an appropriate level of expertise to state that I do not consider it to be of an acceptable scientific standard, for reasons outlined above.

# Peer review: Reviewer response for version 1

**DOI:** 10.21956/openreseurope.14410.r27061

**Published:** 2021-06-29

**Authors:** Elizabeth Pollitzer

**Affiliations:** 1 Portia gGmbH, London, UK

There are a number of potentially promising aspects raised in the paper, namely devising and implementing gender equality policies in engineering education contexts and in countries and institutions with cultural and possibly legal barriers to gender equality, and the experience of the RMEI network functioning as a CoP to advance good practice for gender equality in engineering. Focusing on these topics would be very useful to anyone planing a proposal for Horizon Europe.

 However, these aspects get lost in the current text, which includes description of the TARGET project and extensive discussion of topics only partly relevant to the three questions listed in the Methods issues, e.g., discussion of SDGs.  The SFL is extensively discussed as a framework but only very briefly how it was actually practiced by the CoP for development of gender equality policy for the institutions involved and how the toolbox was developed and tested. I would caution about the description of the leadership style as a combination of 'soft leadership'  and 'female leadership'. Whilst women leaders have been shown often to adopt different leadership styles to men, there is no such a thing as "female" leadership.  I would also caution about the potential misunderstanding of the acronym  "GEP" which is widely used to stand for Gender Equality Plans rather than as in the paper for gender equality policy.

If applicable, is the statistical analysis and its interpretation appropriate?

No

Is the study design appropriate and is the work technically sound?

Partly

Is the work clearly and accurately presented and does it engage with the current literature?

Partly

Are the conclusions drawn adequately supported by the results?

No

Are sufficient details of methods and analysis provided to allow replication by others?

Partly

Are all the source data and materials underlying the results available?

Partly

Reviewer Expertise:

gender equality and gender dimension is science research

I confirm that I have read this submission and believe that I have an appropriate level of expertise to state that I do not consider it to be of an acceptable scientific standard, for reasons outlined above.

# Response

**Published:** 2021-10-05

**Authors:** ANASTASIA ZABANIOTOU

**Affiliations:** Aristotle university of Thessaloniki Greece, Greece

***Reply to comments of REVIEWER #3 Dr Elizabeth Pollitzer, Director, Portia Ltd, UK; founder, Gender Summit***.

**Comment 1:** *There are a number of potentially promising aspects raised in the paper, namely devising and implementing gender equality policies in engineering education contexts and in countries and institutions with cultural and possibly legal barriers to gender equality, and the experience of the RMEI network functioning as a CoP to advance good practice for gender equality in engineering. Focusing on these topics would be very useful to anyone planning a proposal for Horizon Europe.*

**Reply 1: **Thank you for appreciating my effort and taking the time and effort to review my paper.

**Comment 2:** *However, these aspects get lost in the current text, which includes a description of the TARGET project and extensive discussion of topics only partly relevant to the three questions listed in the Methods issues, e.g., discussion of SDGs.* 

**Reply 2:** The reviewer’s comments have been taken into consideration and the paper has transformed to a **METHOD paper,** that builds upon a use case that is the case of the RMEI network where the SFL was implemented and validated, following the suggestion of ORE’s editors. According to ORE guidelines “ ***Method articles*** *in some areas of research, e.g. education, social sciences or economics, may benefit from a different structure, in which case a more flexible format is possible as long as the authors ensure that they describe their methods and sources in sufficient detail for others to be able to repeat the research’. *For this reason, the title has changed to: ***Learning outcomes of leader and leadership development: a method for a soft and female leadership (SFL) empirical framework for driving the gender equality change in Mediterranean engineering education [version 3]***

**Comment 3:** *The SFL is extensively discussed as a framework but only very briefly how it was actually practised by the CoP for the development of gender equality policy for the institutions involved and how the toolbox was developed and tested*.

**Reply 3:** In this revised METHOD paper, in the conclusion part, I added a USE CASE section where it is explained how the SFL is practiced and validated. The SFL was validated at the network level. It is expecting that by implementing the SFL framework members of the RMEI CoP can integrate gender equality knowledge via mindful awareness, widen their experience, deepen their understanding, and build a gender-sensitive mindset. With practice, a journey of looking inward can begin for the participants, with the help of science, art, and culture, via workshops to inquire, challenge, and furthermore proceed with gender equality change at their institution. It is expecting that change agents and new leaders for gender equality will be developed to inspire other higher education institutions and networks to undertake a participatory path towards a gender equality change.

**Comment 4:** *I would caution about the description of the leadership style as a combination of 'soft leadership' and 'female leadership'. Whilst women leaders have been shown often to adopt different leadership styles to men, there is no such a thing as "female" leadership.*

**Reply 4:** Thank you for the comment. At the RMEI, we decided to call the new leadership SFL because it is a combination of a female leader and soft competence building. This is a new term we applied in our method. The specific techniques that are used in the current study are empirical, supported also by the literature, and the gender equality knowledge built by the EU HORIZON2020 TARGET project. The author replicated the soft leadership framework developed by prof Rao M.S  but also added to the field by examining aspects of the engineering education domain and the Mediterranean community of practice (CoP) for gender equality.

**Comment 5:** *I would also caution about the potential misunderstanding of the acronym «GEP" which is widely used to stand for Gender Equality Plans rather than as in the paper for gender equality policy.*

**Reply 5:** Thank you, it is corrected as suggested.

# Peer review: Reviewer response for version 1

**DOI:** 10.21956/openreseurope.14410.r27064

**Published:** 2021-06-23

**Authors:** Monia Chouari

**Affiliations:** 1 Faculty of Arts and Human Sciences, University of Sousse, Sousse, Tunisia

Built upon an interrogation of the dominant gender inequality in higher education institutions, this paper addresses the concept of leader and female leadership in the 21 ^st^ century as a pathway to establish a new promising and creative mindset. To come to terms with such a sublime aim, the paper presents a logical analytical process that falls into five main parts:

1. The introduction including definitions of the key concepts of soft skills and leadership that Figure 1clearly illustrates. Then, it underpins the aim and the methods of the study. In line with the scientists’ assumption about the limits of the concepts of leadership, the author argues that leadership is a social construct that embodies new relationships combining soft skills with women’s leadership ability.
  Leadership integrates context-based characteristics and comprises soft skills foreshadowing a change in the cultural frame of mind rather than focusing on mere reassignment and/ or redistribution of tasks.
  Given that the aim is to affect people’s behaviour(s), manners and ways of modernized thinking to establish women's leadership, the author’s title highlights a complex framework of soft female leadership to boost gender equality in engineering education, as well as in other fields.
2. This study positions itself in the TARGET project that supports GEPs within the network of RMEI that inspires the author’s vision of gender equality via leadership and its diverse performances in various contexts with varied characteristics as mentioned in figure 1. Therefore, the author fairly explains that leadership transcends the conventional set of criteria to focus more profoundly on the mental and affective processes that instigate a new set of behaviour and ethics as an alternative to the existing ones.
3. A thorough investigation of SFL endorsed by a multidimensional gender equality learning process to build up individual and collective behaviour(s) with a far-reaching impact that blurs the conventional borderlines of gender.
  At this level, I applaud the coherent analysis along with the smooth shift from the conceptualized soft skills leadership to ‘transformative learning’ within the educational and societal contexts. The paper has provided thorough implications of the SL in terms of space (Mediterranean institutions, 28 engineering schools, and societies), time (present and future) and power. When the author proceeds to develop SL ‘’new narratives’, readers are systematically provided with Figure 2.
4. The core of the paper contains 5 pages conducting readers to track the six steps of the SFL framework. ‘’SFL framework new deliveries’’ attempts to reveal the interconnectedness between approaches, narratives, and a selected toolbox entailing the change of the mindset and endorsing flexibility towards the achievement of SDGs.
  Based on the author’s knowledge of the socio-cultural and psychological burden of gender inequality in engineering, she insightfully and minutely develops six steps of soft female leadership comprising leadership in research and innovation that inter-connect diverse fields in academia and industry.
5. The discussion section and the conclusions present the author’s combination of the theoretical, thematic and structural dimensions of gender equality from the perspective of soft female leadership that falls into the column of behavioural theory of leadership; figures 3 and 4 explicitly display.

The paper presents an inspiring contribution to academic research prompting context-based female leadership.  In challenge of the male-dominated institutions, the author concludes that each model of leadership should be tailored according to the diagnosed problem with gender equality. Given the thought-provoking arguments, this paper could originate future interdisciplinary research towards the establishment of soft female leadership in institutions and society equally.

 What I also appreciate is the gender-sensitive language which is strategically avoided. For example, the term female, as a noun, normally implies a set of biological characteristics of femaleness. Yet, in this paper, the term ‘female’ is used as an adjective, as in ‘female leadership,’ to imply the author's awareness of the rhetoric of gender.

 Accordingly, I confirm that I have read the submitted paper, and I confirm that it responds to academic scientific standards. I, therefore, approve that this paper is ready to be indexed.

If applicable, is the statistical analysis and its interpretation appropriate?

Yes

Is the study design appropriate and is the work technically sound?

Yes

Is the work clearly and accurately presented and does it engage with the current literature?

Yes

Are the conclusions drawn adequately supported by the results?

Yes

Are sufficient details of methods and analysis provided to allow replication by others?

Yes

Are all the source data and materials underlying the results available?

Yes

Reviewer Expertise:

English Literature; Gender Studies

I confirm that I have read this submission and believe that I have an appropriate level of expertise to confirm that it is of an acceptable scientific standard.

# Response

**Published:** 2021-10-05

**Authors:** ANASTASIA ZABANIOTOU

**Affiliations:** Aristotle university of Thessaloniki Greece, Greece

Acknowledgements to REVIEWER #2 Prof Monia Chouari, University of Sousse, Sousse, Tunisia:

 I would like to deeply thank Prof Monia Chouari for her appreciation of my work, the time she devoted and the effort to analyze in detail all chapters and aspects of the article. Your positive evaluation adds value to my paper. Thank you very much.

# Peer review: Reviewer response for version 1

**DOI:** 10.21956/openreseurope.14410.r27063

**Published:** 2021-06-21

**Authors:** Jerneja Penca

**Affiliations:** 1 Euro-Mediterranean University, Piran, Slovenia

I am not very familiar with the scope and aim of the journal to which I was invited to review. Thus, I may not be pitching my response at the right level, as I opted to provide a review on the article in light of the questions posed to me and my knowledge of the area I am reviewing.

 The article engages with some of the relevant literature to support the claims made, but it does not make it clear how does it build on that literature, what gaps it fills and how does it contribute to existing knowledge. I wonder if a comprehensive survey of existing literature regarding gender and leadership types has even been made. I also wonder about the extent to which expertise from the disciplines of sociology, gender studies or other social science backgrounds have been involved into the research of this interdisciplinary theme.

 It is not clear what kind of empirical data supports the piece. The authors are clear in stating their commitment (as a group) to promote gender equality in engineering schools and learning for sustainability, and to fight the under-representation of women in leadership and senior positions at the engineering education systems. But we miss the information on what study / research was conducted and to what aim. Rather than being supported by a study, the article comes closer to a manifesto. The content may be convincing and I (as probably many other readers) sympathise with the goal of gender equality in managerial positions and the education machinery, support values proposed and agree with multiple other claims made (e.g. that gender equality strategies are context sensitive or that leadership has typically had a masculine construction.). However, the article would need to significantly more precise in various points to qualify as a research article. As a side note, for analytical purposes, I would suggest writing in slightly longer paragraphs where a certain thought is developed, rather than in one sentence paragraphs (Discussion, Conclusion section)

If applicable, is the statistical analysis and its interpretation appropriate?

No

Is the study design appropriate and is the work technically sound?

Partly

Is the work clearly and accurately presented and does it engage with the current literature?

Partly

Are the conclusions drawn adequately supported by the results?

No

Are sufficient details of methods and analysis provided to allow replication by others?

No

Are all the source data and materials underlying the results available?

Partly

Reviewer Expertise:

NA

I confirm that I have read this submission and believe that I have an appropriate level of expertise to state that I do not consider it to be of an acceptable scientific standard, for reasons outlined above.

# Response

**Published:** 2021-10-05

**Authors:** ANASTASIA ZABANIOTOU

**Affiliations:** Aristotle university of Thessaloniki Greece, Greece

***Reply to comments by REVIEWER #1 Jerneja Penca, Euro-Mediterranean University, Piran, Slovenia***

**Comment *1*** *I am not very familiar with the scope and aim of the journal to which I was invited to review. Thus, I may not be pitching my response at the right level, as I opted to provide a review of the article in light of the questions posed to me and my knowledge of the area I am reviewing.*

**Reply 1:** We are sorry you are not very familiar with ORE. For your information, Open Research Europe's scope covers any scholarly work in a social sciences discipline resulting from studies that have Horizon 2020 funding, regardless of the perceived interest and the extent of novelty. The peer review focuses on whether the paper has academic merit and whether adequate detail has been provided to enable others to reproduce and use the method.  Nevertheless, I would like to thank you for making the effort to provide your review that helped us to upgrade the manuscript as a **METHOD paper**. https://open-research-europe.ec.europa.eu/for-authors/article-guidelines/social-sciences/method-articles#main

**Comment 2:** *The article engages with some of the relevant literature to support the claims made, but it does not make it clear how does it build on that literature, what gaps it fills and how does it contribute to existing knowledge. I wonder if a comprehensive survey of existing literature regarding gender and leadership types has even been made.*  

**Reply 2:**  Following the ORE editors, this paper was transformed into a **METHOD paper** because this type of article was the best fit for our paper.  Therefore, the paper as it is transformed to a METHOD paper builds upon a use case that is the case of the RMEI network where the SFL was implemented and validated.’ *Method articles in some areas of research, e.g. education, social sciences or economics, may benefit from a different structure, in which case a more flexible format is possible as long as the authors ensure that they describe their methods and sources in sufficient detail for others to be able to repeat the research* (see article guidelines at https://open-research-europe.ec.europa.eu/for-authors/article-guidelines/social-sciences/method-articles#main).   

 The reviewers’ comments have been taken into consideration and the paper has been revised accordingly. In this revised version it is presented not as a **METHOD paper** with a use case because it fits better the reviewers’ comments and expectations and the type of work reported. The revised title is: ***Learning outcomes of leader and leadership development: a method for a soft and female leadership (SFL) empirical framework for driving the gender equality change in Mediterranean engineering education [version 3]***

**Comment 3:** *I also wonder about the extent to which expertise from the disciplines of sociology, gender studies or other social science backgrounds have been involved in the research of this interdisciplinary theme.*

**Reply 3:** As is stated in the conclusion section, we countered the lack of knowledge on social change theories; therefore, we used our intuition, creativity, interdisciplinarity approaches, and our embedded experience from scientific and academic work, engineering problem-solving methodologies and R&I approaches. All these are complemented with tailored and customized activities, under a reflexive prism that the TARGET project taught us. That’s why the SFL and leaders’ development framework for gender equality advancement in engineering, that we developed at RMEI, is grounded on empirical evidence. It derives as an outcome from an engineering context across Mediterranean countries.

**Comment 4:** *It is not clear what kind of empirical data supports the piece. The authors are clear in stating their commitment (as a group) to promote gender equality in engineering schools and learning for sustainability, and to fight the under-representation of women in leadership and senior positions at the engineering education systems. But we miss the information on what study/research was conducted and to what aim. Rather than being supported by a study, the article comes closer to a manifesto. The content may be convincing, and I (as probably many other readers) sympathize with the goal of gender equality in managerial positions and the education machinery, support values proposed and agree with multiple other claims made (e.g. that gender equality strategies are context-sensitive or that leadership has typically had a masculine construction.). However, the article would need to be significantly more precise in various points to qualify as a research article.* 

**Reply 4:** I fully understand your claims because as you reviewed it as a research paper. Now, that I am resubmitting it as a METHOD paper, I hope you are satisfied with the information provided to support a METHOD paper at ORE. Discussion is now enriched with a section ‘USE CASE’ where the implementation of the SFL is explained.

**Comment 5:** *As a side note, for analytical purposes, I would suggest writing in slightly longer paragraphs where a certain thought is developed, rather than in one sentence paragraphs (Discussion, Conclusion section)*  

**Reply 5:** Following your suggestion, I tried as much as possible, to shorten sentences and paragraphs (Results, Conclusion) and somehow the discussion part.
